# Pediatric craniospinal irradiation with a short partial-arc VMAT technique for medulloblastoma tumors in dosimetric comparison

**DOI:** 10.1186/s13014-020-01690-5

**Published:** 2020-11-05

**Authors:** Gerhard Pollul, Tilman Bostel, Sascha Grossmann, Sati Akbaba, Heiko Karle, Marcus Stockinger, Heinz Schmidberger

**Affiliations:** Department of Radiation Oncology, University Medical Center Mainz, Langenbeckstraße 1, 55131 Mainz, Germany

**Keywords:** Pediatric radiation, Medulloblastoma, Craniospinal irradiation, Partial VMAT

## Abstract

**Background:**

This study aimed to contrast four different irradiation methods for pediatric medulloblastoma tumors in a dosimetric comparison regarding planning target volume (PTV) coverage and sparing of organs at risk (OARs).

**Methods:**

In sum 24 treatment plans for 6 pediatric patients were realized. Besides the clinical standard of a 3D-conformal radiotherapy (3D-CRT) treatment plan taken as a reference, volumetric modulated arc therapy (VMAT) treatment plans (“VMAT_AVD” vs. “noAVD” vs. “FullArc”) were optimized and calculated for each patient. For the thoracic and abdominal region, the short partial-arc VMAT_AVD technique uses an arc setup with reduced arc-length by 100°, using posterior and lateral beam entries. The noAVD uses a half 180° (posterior to lateral directions) and the FullArc uses a full 360° arc setup arrangement. The prescription dose was set to 35.2 Gy.

**Results:**

We identified a more conformal dose coverage for PTVs and a better sparing of OARs with used VMAT methods. For VMAT_AVD mean dose reductions in organs at risk can be realized, from 16 to 6.6 Gy, from 27.1 to 8.7 Gy and from 8.0 to 1.9 Gy for the heart, the thyroid and the gonads respectively, compared to the 3D-CRT treatment method. In addition we have found out a superiority of VMAT_AVD compared to the noAVD and FullArc trials with lower exposure to low-dose radiation to the lungs and breasts.

**Conclusions:**

With the short partial-arc VMAT_AVD technique, dose exposures to radiosensitive OARS like the heart, the thyroid or the gonads can be reduced and therefore, maybe the occurrence of late sequelae is less likely. Furthermore the PTV conformity is increased. The advantages of the VMAT_AVD have to be weighed against the potentially risks induced by an increased low dose exposure compared to the 3D-CRT method.

## Background

With an incidence of 18–20% of all brain tumors, medulloblastoma are the most common type of malign brain tumors in childhood [1, 2]. As one part of the curative treatment approach in a combined-modality, it is recommended to irradiate the whole brain and the entire craniospinal axis [3]. The 5 year event-free survival (EFS) rate nowadays obtains values exceeding 80% for children older than three years with nondisseminated disease for concomitant radiochemotherapy (Medulloblastoma Average Risk; Cisplatin/Cyclophosphamide) [4, 5]. However, it has been turned out that any kind of radiotherapy combined with chemotherapy in childhood is a risk factor for the development of several late complications [6]. Especially neuropsychological, neurological, ophtalmological and endocrine disorders are corresponding sequelae [7]. Additionally, cranial irradiations are often associated with declines in academic ability, social skills and attention [8].

Moreover, recent studies like the CCSS (Childhood Cancer Survivor Study), the CVSS (Cardiac and Vascular late Sequelae in long-term Survivors of childhood cancer) or the RISK register (Register of Treatment-Associated Late Effects after Radiotherapy of Malignant Diseases in Childhood and Adolescence) have shown a substantial increased presence of cardiovascular diseases as a late sequelae from initial irradiation in childhood. Furthermore, metabolic diseases or a radiation-induced hypothyroidism often arise [9–12]. In terms of female patients treated with ionizing radiation, late effects can occur relating to acute ovarian failure for minimal dose exposures > 10 Gy. This can result in premature nonsurgical menopause or a limited probability for pregnancy [13].

Conventionally the radiation therapy for the brain and the entire craniospinal axis is done with the 3D-conformal radiotherapy. These body regions are treated with two opposing lateral beams adjusted to one posterior beam. Thus it is necessary to kick the couch by 90° to match the diverging beams in between the brain and the spine [14, 15]. The high sensitivity to field matching errors can lead to a dosimetric heterogeneity and uncertainty in the planning target volume and determine the need for feathering of junctions [16, 17]. For a VMAT planning with an overlapping area of field junction, the dose distribution is much less sensitive to the uncertainties caused by mechanical inaccuracies or patient setup errors [18]. Another issue is the dose deposition anterior to the spinal target volume due to the single posterior irradiation field, which leads to considerable dose accumulations in organs at risk like the heart, the thyroid and the intestines [19].

For this reasons there are several trials to reduce the likelihood for the incidence of organic damage. Considering that some late sequelae eventually result from too high doses to the corresponding organs, one approach is to diminish the total dose for the spine from 36.0 to 23.4 Gy, supplemented by a local tumor dose of 31.8 Gy to the posterior fossa (total dose of 55.2 Gy). In this regard, several studies have already reported comparable 5-year survival rates [19–21].

A further approach is using modern irradiation techniques for a better dose-sparing in OARs and a more conformal application [6, 22]. Several methods have been developed to improve the optimization capabilities [3, 23–25]. However the evidence is not clear to the rareness of data.

In this study treatment plans with the VMAT method were realized with a short partial-arc length setup and the use of “avoidance sectors” referred to as “VMAT_AVD”. This setup was developed to reduce dose to OARs to be located anterior to the spine and to reduce low-dose exposure to the lungs simultaneously.

Intensity modulated treatment plans mostly use more beam directions and almost always have an increased number of monitor units compared to 3D-CRT. This leads to a raised amount of scatter dose with the effect of a larger volume accumulating low-dose. This may result in a greater probability for the incidence of secondary malignancies [26]. This uncertainty is still a part of actual investigations and has mostly unexplained yet [25, 27].

In this study 6 patients with the diagnosis of medulloblastoma were included, who were initially treated with a 3D-CRT technique, with the aim of a retrospective plan comparison using the VMAT_AVD technique. The main focus lies on the comparative analysis of dose exposures to organs at risk and the comparison with the current literature to present also additional information. Furthermore the VMAT_AVD trial has been evaluated against setups using more beam directions.

## Patients and methods

### Patients

The patient collective includes 6 children with an average age of 9 years (range 5–16 years). Tumorectomy has already been performed before the start of radiotherapy. Total planning target volume (PTV) extends over a length from 53 to 74 cm. This requires the usage of at least two isocenters for irradiation techniques via common linear accelerators, independent of the choice of the treatment method.

### Patients positioning

All patients were CT-scanned in supine position with purpose-built, individual thermoplastic masks, shaped for the head with five-point fixation to achieve an adequate immobilization and also a good reproducibility during treatment. Due to the retrospective trial with a changed beam setup, the arms were excluded for the most part of the body outline structure. Originally, the patient position on the CT scan was arms rested beside body left and right for the simple reason, that the classical 3D-conformal irradiation technique does not include lateral beams for the spine. With arms lying on the body and hands on femurs an irradiation from the side direction should be possible without any disadvantages in patient positioning.

### Contouring and dose calculation

The contouring on the image sets consists of various OARs like the lens, parotid glands, lungs, esophagus, thyroid, heart, liver, kidneys and the gonads. In addition, the cranial part of the CTV (Clinical Target Volume), including the entire brain, cranial nerves and meninges, was defined. The caudal part of the CTV comprises the entire subarachnoid space from the foramen magnum to the lower limit of the thecal sac plus an extension laterally to include the nerve roots analogous to the “SIOPE guideline” [28]. The CTV to PTV margin in the spine region was set to 5 mm circular.

Treatment planning for the 3D-CRT and the VMAT plans was realized with the treatment planning system (TPS) “Eclipse” from Varian (Varian Medical Systems, Palo Alto, CA, USA) and calculated with the “Anisotropic Analytical Algorithm” (AAA) Version 13.026. The linear accelerator beam model used for calculation is a “TrueBeam Vers.2.5” from Varian. For the dose optimization process, the “Progressive Resolution Optimizer” (PRO) Version 13.026 from Varian was selected. All treatment plans were prescribed to a dose of 35.2 Gy (22 fractions, 1.6 Gy per day) which in most cases corresponds to the initial irradiated dose with 3D-CRT.

### 3D-CRT treatment planning

All 6 patients have initially been treated with a 3D conformal irradiation plan. These plans consist mainly of two lateral beams for the brain region with a few degrees of collimator and couch rotation. The beams are matched plane-parallel to a posterior beam for the spine with a gantry rotation of approximately 170° and a couch kick of 90°. Gantry rotation by approximately 180°–10° was used to avoid too much face-dose accumulation. Thus, the jaw matching to the brain fields will be nearly orthogonal to the couch and head position. Additionally, several subfields were generated to take into account different location of vertebral body in terms of depth from skin. The used Source to Skin Distance (SSD) of the posterior beam depended on PTV length and was adapted accordingly. The treatment plans contain always at least two isocenter positions. For one patient with the largest PTV length of 74 cm, two different located isocenter positions for the spine were necessary. The original 3D-CRT treatment plans were adapted to a standardized PTV in terms of margins, based on the rarely modified CT scans described above and were recalculated with the latest version of the dose calculation algorithm. Dose constraints were focused on spine and brain within 95 and 107% of the prescription dose.

### VMAT treatment planning

**Fig. 1:**
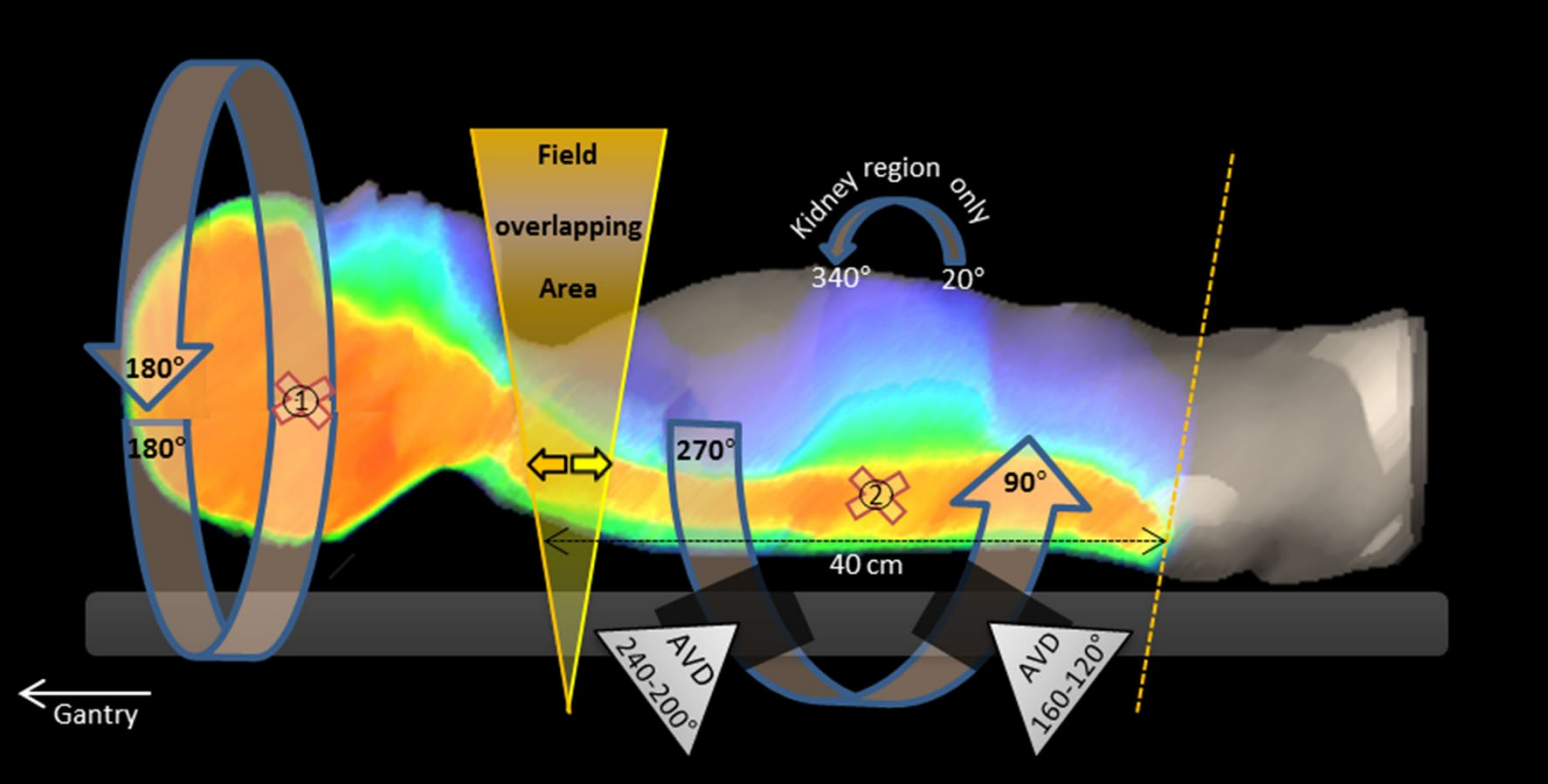
Beam setup for the VMAT_AVD technique in detail. For the cranial brain and neck PTV a full 360° rotation (isocenter 1) and for the caudal spine PTV a half rotation from 90° to 270° (isocenter 2) were applied. Avoidance sectors “AVD” of 40° (no irradiation) were applied (black area). For the kidney region limited anterior irradiation was used. The field overlapping area with the corresponding jaw positions is highlighted in yellow. The isocenter positions are marked with a red cross

Retrospectively for all 6 patients treatment plans using a VMAT technique, named “VMAT_AVD” were generated. The always two isocentric based plans consist of three 360° arc beams with different collimator and jaw positions for the cranial part of the PTV and upper spine-PTV. For the remaining spine-PTV in sum 6 quarter arcs including 40° avoidance sectors were used from 180° to 90° and from 180° to 270°, shown in Fig. 1. Avoidance sectors were set to reduce the volume of normal tissue exposed by low dose. Due to the always large field size close to 40 cm, collimator rotation was set to only a few degrees. The overlapping area between the fields of the two isocenters varies in between 2 and 4 cm to reduce sensitivity for uncertainties and was placed according to PTV’s length in the maximum cranial position (40 cm field size).

Mostly for all contoured OARs dose optimization constraints for the inverse planning process were set for each patient individually and also a normal tissue objective with low priority was used to force the decline of dose outside the target volume. Close attention was paid on organs like the heart, thyroid, gonads, kidneys and lungs (V5, V10, and V20Gy). The highest priority during the optimization process was set to the spine and the brain receiving at least 95% of the prescription dose with minimizing the dose greater than 107% simultaneously. Planning objectives used for optimization differ from each patient due to individual characteristics and therefore have been stated only exemplarily in the appendix (please see Additional File 1: Table S1). There is only a fixed movement vector in the longitudinal and vertical direction from one isocenter to the other.

To explain the decision for the applied beam setup, alternative treatment plans in VMAT technique were also generated and presented in a DVH-comparison (Dose Volume Histogram) referred to the lungs, the body outlines and the breasts. One modified setup uses the same beam setup except the avoidance sectors called “noAVD” and additionally another modified setup uses full 360° rotations, called “FullArc” (only theoretical; position of the arms not considered). Comparisons between the three VMAT techniques are illustrated in the appendix.

### Evaluation

For several OARs and the PTVs the DVH’s were compared for the different treatment methods. Mostly mean doses and especially for the lungs different volume characteristics like the V5, V10 and V20 (Volume in percent receiving dose of 5, 10 and 20 Gy) were evaluated.

As a quality treatment planning tool the dose conformity referred to the PTV was also determined using the dose conformity index (CI) by Eq. (1).

1 $${\raise0.7ex\hbox{${CI = \left( {V_{T,RI} } \right)^{2} }$} \!\mathord{\left/ {\vphantom {{CI = \left( {V_{T,RI} } \right)^{2} } {\left( {V_{T} *V_{RI} } \right)}}}\right.\kern-\nulldelimiterspace} \!\lower0.7ex\hbox{${\left( {V_{T} *V_{RI} } \right)}$}}$$

*V*_T,RI_ is the volume of the PTV covered by reference isodose line of 95%, based on ICRU report [PTV be confined within 95–107% of the prescribed dose (ICRU, 1999)]. *V*_T_ is the volume of the PTV and *V*_RI_ the volume of the reference isodose [29].

## Results

**Fig. 2:**
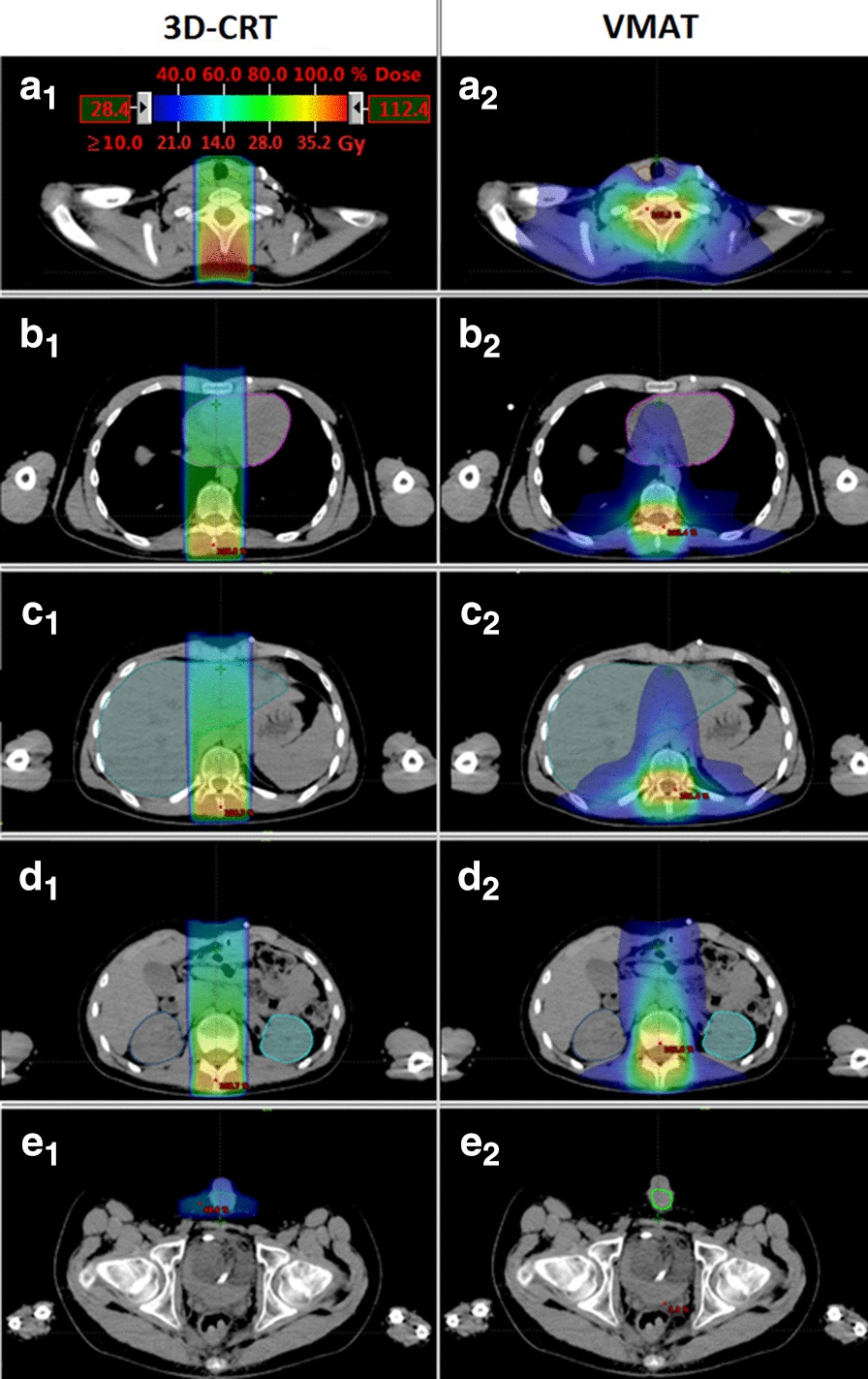
Transversal dose distributions for different regions of the spine PTV with a lower dose threshold of 10 Gy (28.4%) for the 3D-CRT (left) and VMAT (right) technique. Representative parts of the body are selected: **a** lower neck region; **b** mid-thoracic region; **c** upper abdominal region; **d** mid-abdominal region, **e** lower pelvic region

Figure 2 shows transversal dose distributions for different regions of the body for VMAT_AVD and 3D-CRT in comparison.

The resulting dosimetric differences for several OARs are highlighted in Table 1. For VMAT_AVD the mean doses especially for the heart, thyroid and gonads are reduced from 16 to 6.6 Gy, from 27.1 to 8.7 Gy and from 8.0 to 1.9 Gy for the heart, the thyroid and the gonads respectively, compared to the 3D-CRT treatment method. The mean doses to the liver, the body outlines and the breasts are comparable with VMAT_AVD and 3D-CRT. In Addition the mean doses for the lungs and the kidneys are increased by 3.3 and 2.7 Gy for VMAT_AVD. For the spinal cord as part of the PTV, the calculated dose depositions are almost identical. Especially noteworthy is the increased volume receiving higher doses for 3D-CRT compared to VMAT_AVD (for example, V110% body outline: 149 ccm vs. 0.9 ccm). This also affects the dose conformity with lower values for the 3D-CRT (Table 2). For the comparison with VMAT_noAVD and VMAT_FullArc, please see Additional File 2: Table S2 and Addtional File 3: Table S3.

**Table 1:** Dosimetric comparison between VMAT_AVD and 3D-CRT

Technique	VMAT_AVD	3D-CRT	Diff. abs	
OARs	D_mean_ [range]	D_mean_ [range]	Δ in Gy	Ratio in %
Heart	6.6 [5.8–7.0]	16.0 [13.7–18.5]	− 9.4	41.3
Thyroid	8.7 [7.6–9.9]	27.1 [24.9–29.5]	− 18.4	32.1
Lungs	7.5 [7.0–7.9]	4.2 [2.8–5.3]	3.3	178.6
Kidneys	5.3 [4.7–5.8]	2.6 [1.7–3.9]	2.7	203.8
Liver	5.7 [5.2–6.3]	4.8 [4.7–7.4]	0.9	118.8
Breast	2.4 [2.0–3.2]	2.1 [1.4–2.4]	0.3	114.3
Spinal cord	35.5 [35.2–35.9]	35.3 [34.9–35.8]	0.2	100.6
Body outline	11.5 [9.9–12.7]	11.1 [8.5–13.2]	0.4	103.6
Gonads^a^	1.9 [0.4–4.3]	8.0 [1.3–25.1]	− 6.1	23.8
Gonads D_max_	3.9 [0.7–10.4]	17.5 [3.5–28.6]	− 13.6	17.1

*VMAT_AVD *volumetric modulated arc therapy with avoidance sectors, *3D-CRT *3D-conformal radiotherapy, *Diff. abs.* absolute dose difference, *D*_*mean*_ mean dose in Gy, *D*_*max*_ maximum dose in Gy

^a^The localization for the ovaries is uncertain due to lack of MRT data; therefore it is rather a rough indication

**Table 2:** Conformity indices VMAT_AVD versus 3D-CRT

Technique	VMAT_AVD	3D-CRT
Conformity index^a^	CI	CI
Patient 1	0.84	0.51
Patient 2	0.85	0.70
Patient 3	0.90	0.74
Patient 4	0.89	0.67
Patient 5	0.88	0.71
Patient 6	0.83	0.53

*VMAT_AVD* volumetric modulated arc therapy with avoidance sectors, *3D-CRT* 3D-conformal radiotherapy

^a^Conformity index calculated using methodology by Riet et al. [25]

**Fig. 3:**
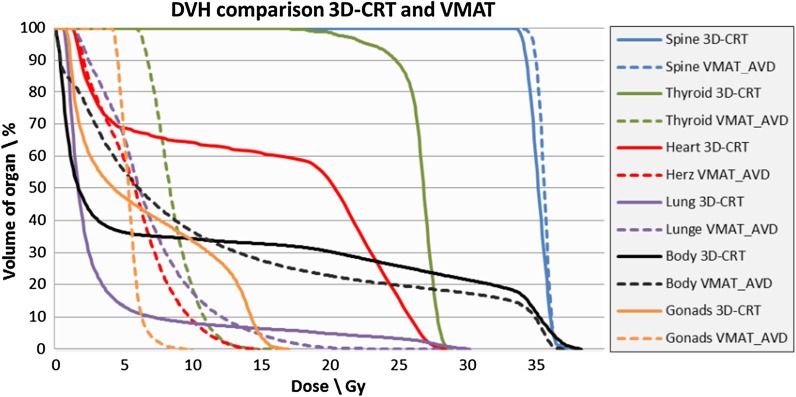
Dose volume histogram comparison between VMAT (dashed line) and 3D-CRT (solid line) for several relevant organs exemplary shown for one patient. The dose reduction for the thyroid and heart (green and red lines) is obvious. Different curves for the lungs in terms of high or lower values V20, V10 and V5Gy are comprehensible (violet lines)

For better illustration the dosimetric differences for the individual organs are presented in a DVH for one patient (Fig. 3). Due to the increased low-dose exposure in patients’ bodies, the volumes receiving 5 Gy (V5) and 10 Gy (V10) for the lungs are increased, with values of 65.2 and 24.8% compared to 16.0 and 10.4% for VMAT_AVD and 3D-CRT respectively. Nevertheless, for higher doses there is a break-even point in between 10 and 20 Gy. Contrary to low-dose exposures, using VMAT_AVD leads to a decreased lungs volume receiving 20 Gy by 5% difference (Table 3).

**Table 3:** Dose statistics shown for the lungs

Technique	VMAT_AVD	3D-CRT	Δ in %
Lungs	Vol%	Vol%	Vol%
V5Gy	65.2 [61.0–70.8]	16.0 [8.3–21.3]	49.2
V10Gy	24.8 [20.9–28.9]	10.4 [4.8–15.0]	14.4
V20Gy	2.0 [1.7–2.5]	7.0 [2.8–10.3]	− 5.0

*V5Gy* lungs volume receiving doses more than 5 Gy, *VMAT_AVD* volumetric modulated arc therapy with avoidance sectors, *Vol%* percentage of volume

Compared to the partial arc setups, the use of a full rotation VMAT setup leads to a further enlarged volume low-dose exposed, with a V5Gy of 92.1% (please see Additional File 4: Table S4).

In Addition these differences for low dose exposures are shown in a DVH-comparison for the three different VMAT setups (see Additional File 5: Figure S5).

By comparing dosimetric results for selected organs, Table 4 give an overview for consideration in the context of existing literature, dealing also with craniospinal irradiation.

**Table 4:** Mean doses given in Gy for OARs with VMAT in comparison, normalized to 35.2 Gy

	This study	Myers et al. [30]	Seravalli et al. [25]^a^	Parker et al. [31]	Myers et al.	Seravalli et al.
CTV-PTV margin spine in mm	5.0 circular	7.0 circ.^‡^	5.0 circ	3.0 circ.^‡^	7.0 circ.^b^	5.0 circ
N [age]	6 [5–16 y]	24 [2–18 y]	1 [14 y]	1 [19 y]	24 [2–18 y]	1 [14 y]
No. of isocenters	2	2	3	1	1	1
Partial arc + AVD	Yes	No	Yes	No	No	No
Organ/technique	VMAT_AVD	VMAT	VMAT	HT	HT	HT
Heart	6.6 [5.8–7.0]	6.6 ± 1.1	6.8 [5.6–10.7]	10.8 [N.A]	7.1 ± 1.1	9.2 [7.5–11.6]
Thyroid	8.7 [7.6–9.9]	12.8 ± 1.2	12.7 [5.5–24.1]	24.4 [N.A]	11.3 ± 1.8	15.0 [6.8–19.3]
Lungs	7.5 [7.0–7.9]	9.6 ± 1.4	8.9 [7.9–9.8] ^c^	5.9 [N.A]	8.2 ± 1.4	8.0 [7.0–8.7] ^c^
Kidneys	5.3 [4.7–5.8]	7.8 ± 0.9	6.4 [5.6–8.5] ^c^	6.4 [N.A]	7.9 ± 1.7	6.1 [5.2–6.5] ^c^
Liver	5.7 [5.2–6.3]	6.8 ± 0.9	N.A	7.8 [N.A]	6.6 ± 0.6	N.A

*VMAT_AVD* volumetric modulated arc therapy with avoidance sectors, *circ.* circular, *y* years, *N* number of patients, *No.* number of, *N.A.* data not available, ± given SD., *[w.x–y.z]* range, *HT* helical tomotherapy

^**a**^Values are median mean dose

^b^CTV = spinal cord without nerve roots

^c^Average of right and left organ part

## Discussion

With the VMAT_AVD technique, the dose exposure to OARs like thyroid, heart and ovaries could be reduced compared to the 3D-CRT. Childhood cancer survivors received radiotherapy are at a greater risk of developing hypothyroidism. Furthermore the risk increases with the total irradiation dose to the organ [32]. Verlooy et al. [33] reported an incidence of 43.1% thyroid induced endocrine sequelae in a study from “la Société Française d'Odontologie Pédiatrique” examining former treated medulloblastoma patients in childhood. Also for medulloblastoma patients, high pathological thyroid values were reported by Bolling et al. with a frequency of 56% after a median time of 35 months [9]. Therefore dose reduction to the thyroid may be one major key to reduce or prevent critical late toxicity.

In a retrospective analysis, spinal irradiation in childhood was also associated with a significant risk of developing cardiac dysfunction. Furthermore this relationship was correlated to only a relatively small volume of the heart within the radiotherapy field [34]. Previous data on this topic is mainly based on irradiation techniques, using only posterior beams for the spine and the dose deposition to the heart in average is often about half of the prescription dose. Depending on field expansion in lateral direction, partial parts of the heart are directly located within the treatment field and receive even higher doses. In an analysis of the “German CVSS-study”, including 1002 childhood cancer survivors, an increased risk for premature cardiovascular disease in comparison to three population samples was revealed [11]. Often these dysfunctions occur late, even many years after treatment. In 21% of childhood rhabdomyosarcoma cancer long-term survivors, photon irradiation resulted in one or more cardiopulmonary sequelae [35]. The reduction of the heart mean dose and the volume accumulating middle or high dose, will decrease irradiation-induced cardiac dysfunctions very likely.

In this study the localization of the ovaries is uncertain and a fusion with MRI data is recommended [36]. However, the potential of decreasing the dose in the region of gonads could be demonstrated with regard to average and also to high dose. The number of viable ovarian primordial follicles is reduced after irradiation. This may lead to premature ovarian failure and subsequently to sterility [37]. With intensity modulated radiotherapy (IMRT) the ovarian dose can be reduced [38]. Additionally, Green et al. reported an acute ovarian failure after irradiation in childhood with an incipient risk factor for exposure of high-dose-irradiation > 10 Gy [13]. For the 3D-CRT treatment plans, the maximum doses are often higher than 10 Gy (4 of 6 patients) in contrast to mostly lower values well below 10 Gy for the VMAT_AVD treatment plans (5 of 6 patients). Due to irradiation from posterior with a 90° couch kick and approximately 170° gantry rotation clockwise, wider beam divergence leads to dose accumulation in distant lower pelvis regions caudal to the PTV.

Organs like the heart or the breasts are some kind of antagonists in terms of lungs dose-sparing. For a lower dose deposition in these organs, more lateral irradiation is necessary, which leads to higher dose exposure of the lungs. In the International Project on Prospective Analysis of Radiotoxicity in Childhood and Adolescence (IPPARCA) based on the “RiSK registry”, the authors ascertain a similarity between adult and children in terms of lungs-irradiation toxicity. For lowering the risk of toxicity they recommend to keep the lungs doses for V5Gy, V10Gy, V15Gy and V20Gy as low as possible (e.g., at least V5Gy < 50%, V10Gy and V15Gy < 35% and V20Gy < 30%) [39]. Except the amount of volume receiving 5 Gy, these limits have been complied with the VMAT_AVD technique. In comparison to VMAT setups using more beam projections (noAVD) or even 360° rotations (FullArc), especially the V5Gy values increase up more than 90%. For that reason the VMAT_AVD method uses avoidance sectors of 40° each from posterior to lateral directions, to minimize the V5Gy.

Long-term survivors experience a wide spectrum of radiation-related late effects. These effects include endocrine deficiencies, cardiomyopathy, impaired fertility and the occurrence of second malignancies. Most of these late effects are dose- and volume-related [2, 4, 5]. Therefore dose reduction in critical organs could be one promising approach.

Due to an increased number of cancer survivors, the occurrence of second cancers has raised from 9% of all cancer diagnoses in 1975–1997 to 19% in 2005–2009 [40]. In a study on the basis of data from the “GCCR” (German Childhood Cancer Registry), a cumulative incidence of second malignancies after surviving childhood cancer over all entities is about 8.3%, referred to a follow-up interval up to 35 years [41]. With long-term follow-up studies, the incidence of radiation-induced secondary malignancies after conventional craniospinal irradiation has been reported to be 4.2% after 10 years in the “Children’s Oncology Group A9961 study” and 4.3% 4.3–11.8 years after primary surgery [26, 42, 43].

Regarding the body outline structure, the mean dose accumulation for all patients was similar for both treatment planning methods. Nevertheless, the amount of tissue receiving low dose is increased by using VMAT. Due to more beam directions and scattering, almost the entire body located around the PTV is at least low-dose-affected.

Children have an increased risk to develop radio-induced secondary cancer and a correlation to age has been reported. In addition there seem to be a nearly 3–6 times higher sensitivity to carcinogenic effects of radiation for children compared to adults [44]. Furthermore due to the thyroid is a highly radiosensitive organ there is also an increased risk for the development of a secondary cancer, especially for females after irradiation in childhood [46].

A much discussed assumption is that an increased low-dose exposure in large volumes of the body also results in a higher risk for the occurrence of secondary cancer [23, 26, 46]. This possible causality has not been proved yet and one explanation is the lack of long-term follow-up data for pediatric patients treated with intensity modulated irradiation techniques [26, 27]. For this reason Holmes et al. used a mathematical model to provide a reasonable estimation of risk for second malignancies. This model uses data available at time of treatment planning and is based on an approach by Shuryak et al., unifying short- and long term models [26, 47]. The results in comparing HT with 3D-CRT in terms of developing second malignancies, point out a low risk for both techniques in general. First exception was found for the breasts, with a much higher risk for treatment plans in HT technique. Also a slightly increased and decreased risk has been turned out for the lungs and thyroid, respectively [26]. In contrast, other authors reported that HT does not seem to increase the integral dose to patients’ bodies for very young and small children compared to 3D-CRT [23]. Also, in a report of second cancer risk in a cohort of childhood cancer survivors treated with IMRT, Casey et al. [48] did not find an enhanced rate of second cancer compared to conventional radiotherapy.

Studies based on 3D-CRT treatment plan data for medulloblastoma revealed an occurrence of secondary tumors either within the irradiation beam or in regions affected at least by scatter dose [43]. Frequently arising of second tumors in regions accumulated middle or high doses in between 20 and 30 Gy has also been reported [49]. As mentioned before, contentious to that is the role of modern techniques like VMAT or HT. Maybe less amount of middle or high dose possibly even leads to a decreased incidence of second malignancies [25, 50]. Other authors like Myers et al. [51] even report VMAT or HT techniques for pediatric patients minimize the risk of secondary cancer compared to 3D-CRT. However this is still under investigation has not been proved yet and is still discussed controversial (Additional file 4).

In comparison to already published data dealing with craniospinal irradiation using also VMAT or HT techniques, the achieved dose-sparing for the thyroid is better. However, in a multicenter dosimetric comparison for craniospinal irradiation by Seravalli et al., a wide range in mean doses for organs was reported. Therefore the thyroid mean dose ranges from 5.6 to 24.6 Gy for VMAT techniques due to different intra-institutional planning strategies [25].

Regarding the heart mean dose, the results are also equal or better. Nevertheless, an evaluation of the treatment plan quality for craniospinal irradiation should focus on both, the heart and lungs dose. Compared to the literature, the achieved average mean dose to the lungs is lower in four cases and one trial with smaller CTV to PTV margin result in better dose-sparing for the lungs. The margin, especially in the lateral direction, has a direct impact of dose accumulation in surrounded areas and plays an important role for dose-sparing to OARs. Therefore, lower margins lead to decreased normal tissue irradiation [52]. In addition different kind of contouring, very different patient collectives, varying margin concepts or summarized multi-institutional results complicate the comparability of published study data. So this rather should be understood as a demonstration of optimization capabilities using the presented arc technique and to provide additional information (Additional file 5).

Furthermore several limitations of this study have to be mentioned. First, based on the inclusion of only six patients only descriptive statistics are used. Second, we did not compare our method to IMRT, proton or heavy ion irradiation techniques. Third, the original CT dataset has also been modified and the arms have been excluded. Fourth, the optimization objectives have not been adjusted for the two other VMAT techniques (noAVD + FullArc) and are the same for the VMAT_AVD method. Fifth, the comparison to the literature may not be meaningful in consequence of missing information and values or different patient collectives. However this study should provide additional information for treatment planning methods for medulloblastoma disease due to limited study data so far. Further studies may investigate more individual setup arrangements like subfield arcs or the implementation of deep inspiration setup methods to enlarge the volume of the lungs for better dose sparing.

## Conclusion

Craniospinal irradiation with an optimized VMAT_AVD technique in respect of beam projections could be demonstrated as a feasible alternative to the standard 3D-CRT technique and to 360° full rotation setups. Dose-sparing to the heart and the thyroid and the gonads are the major advantages, besides no junction uncertainness due to setup errors. Especially for lower doses like the V5Gy, this setup arrangement leads to better results compared to irradiation techniques using more beam directions.

## Supplementary information

**Additional file 1: Table S1**. Planning objectives for inverse optimization exemplarily for patient 1.

**Additional file 2: Table S2**. Dosimetric comparison of three VMAT methods.

**Additional file 3: Figure S3**. Conformity indices for three VMAT methods.

**Additional file 4: Table S4**. Dose statistics shown for the lungs for three VMAT methods.

**Additional file 5: Table S5**. DVH comparison for three different VMAT setups.

## Data Availability

The datasets used and/or analyzed during the current study are available from the corresponding author on reasonable request.

## Abbreviations

CT: Computed tomography
CTV: Clinical target volume
PTV: Planning target volume
OAR: Organ at risk
DVH: Dose volume histogram
3D-CRT: 3D conformal radiotherapy
VMAT: Volumetric modulated tadiotherapy
AVD: Avoidance (VMAT including avoidance sectors)
