# Supplementary material for: Pediatric craniospinal irradiation with a short partial-arc VMAT technique for medulloblastoma tumors in dosimetric comparison
Source: Radiat Oncol. 2020 Nov 5;15:256. doi: 10.1186/s13014-020-01690-5 (PMC7643335; doi:10.1186/s13014-020-01690-5)
Supplement: Supplementary file 1 — Additional file 1: Table S1. Planning objectives for inverse optimization exemplarily for patient 1. [file 13014_2020_1690_MOESM1_ESM.docx]

**Additional file 1:**

**Supplementary Table S1:** Planning objectives for inverse optimization exemplarily for patient 1.

| Structure | Limit (Objective) | Vol \ % | Dose \ Gy | Priority |
| --- | --- | --- | --- | --- |
| Normal Tissue | “Automatic sparing” |  |  | 6 |
| Body outline | Upper | 0 | 38.8 | 450 |
| OAR_Heart | Upper | 65.9 | 5.5 | 50 |
| OAR_Heart | Upper | 1.6 | 13.9 | 50 |
| OAR_Heart | Mean |  | 5.3 | 50 |
| OAR_lens | Mean |  | 7.0 | 60 |
| OAR_left_lung | Upper | 60 | 5.0 | 60 |
| OAR_left_lung | Mean |  | 5.8 | 60 |
| OAR_right_lung | Upper | 60 | 5.0 | 60 |
| OAR_right_lung | Mean |  | 6.2 | 60 |
| OAR_kidney (L/R) | Mean |  | 4.5 | 50 |
| OAR_spine | Upper | 0.1 | 35.5 | 150 |
| OAR_spine | lower | 99.9 | 34.4 | 140 |
| OAR_Ovaries | Mean |  | 0.8 | 50 |
| OAR_Liver | Mean |  | 5.0 | 50 |
| OAR_Thyroid | Mean |  | 8.2 | 80 |
| OAR_PTV | Upper | 0.5 | 36.8 | 120 |
| OAR_PTV | Lower | 99.6 | 34 | 150 |
| OAR_PTV | Mean |  | 35.2 | 80 |

**Abbreviations:** OAR = Organ at risk.
