# Supplementary material for: Pediatric craniospinal irradiation with a short partial-arc VMAT technique for medulloblastoma tumors in dosimetric comparison
Source: Radiat Oncol. 2020 Nov 5;15:256. doi: 10.1186/s13014-020-01690-5 (PMC7643335; doi:10.1186/s13014-020-01690-5)
Supplement: Supplementary file 2 — Additional file 2: Table S2. Dosimetric comparison of three VMAT methods. [file 13014_2020_1690_MOESM2_ESM.docx]

**Additional file 2:**

**Supplementary Table S2:** Dosimetric comparison of three VMAT methods.

| Technique | VMAT_AVD | VMAT_noAVD | VMAT_FullArc |
| --- | --- | --- | --- |
| OARs | **D_mean_ [Range]** | **D_mean_ [Range]** | **D_mean_ [Range]** |
| Heart | 6.6 [5.8-7.0] | 6.1 [5.5-6.7] | 6.9 [6.1-7.6] |
| Thyroid | 8.7 [7.6-9.9] | 8.6 [7.7-9.4] | 8.7 [7.8-9.7] |
| Lungs | 7.5 [7.0-7.9] | 7.5 [7.3-7.8] | 8.6 [8.1-9.1] |
| Kidneys | 5.3 [4.7-5.8] | 5.1 [4.6-5.5] | 6.1 [5.7-6.6] |
| Liver | 5.7 [5.2-6.3] | 5.5 [5.2-6.0] | 6.8 [5.2-7.6] |
| Breast | 2.4 [2.0-3.2] | 3.5 [2.7-4.5] | 4.7 [3.9-5.2] |
| Spinal cord | 35.5 [35.2-35.9] | 35.6 [35.3-36.1] | 35.4 [35.3-35.6] |
| Body outline | 11.5 [9.9-12.7] | 11.3 [9.7-12.6] | 12.1 [10.3-13.4] |
| Gonads* | 1.9 [0.4-4.3] | 2.0 [0.5-3.7] | 1.9 [0.5-3.6] |
| Gonads D_max_ | 3.9 [0.7-10.4] | 3.3 [0.8-8.0] | 2.7 [0.8-6.0] |

**Abbreviations:** VMAT_AVD = Volumetric modulated arc therapy with avoidance sectors; VMAT_noAVD = VMAT without avoidance sectors; VMAT_FullArc = VMAT without 360 degrees rotation; D_mean_ = mean dose in Gy; D_max_ = maximum dose in Gy.

* The localization for the ovaries is uncertain due to lack of MRT data; therefore it is rather a rough indication.
