# Supplementary material for: Pediatric craniospinal irradiation with a short partial-arc VMAT technique for medulloblastoma tumors in dosimetric comparison
Source: Radiat Oncol. 2020 Nov 5;15:256. doi: 10.1186/s13014-020-01690-5 (PMC7643335; doi:10.1186/s13014-020-01690-5)
Supplement: Supplementary file 3 — Additional file 3: Figure S3. Conformity indices for three VMAT methods. [file 13014_2020_1690_MOESM3_ESM.docx]

**Additional file 3:**

**Supplementary Table S3:** Conformity indices for three VMAT methods.

| Technique | VMAT_AVD | VMAT_noAVD | VMAT_FullArc |
| --- | --- | --- | --- |
| Conformity Index* | **CI** | **CI** | **CI** |
| Patient 1 | 0.84 | 0.84 | 0.85 |
| Patient 2 | 0.85 | 0.91 | 0.90 |
| Patient 3 | 0.90 | 0.86 | 0.83 |
| Patient 4 | 0.89 | 0.87 | 0.89 |
| Patient 5 | 0.88 | 0.90 | 0.90 |
| Patient 6 | 0.83 | 0.87 | 0.86 |

**Abbreviations:** VMAT_AVD = Volumetric modulated arc therapy with avoidance sectors; VMAT_noAVD = VMAT without avoidance sectors; VMAT_FullArc = VMAT without 360 degrees rotation.

* conformity index calculated using methodology by Riet. et al. [[29](#_ENREF_29)] .
