# Supplementary material for: Pediatric craniospinal irradiation with a short partial-arc VMAT technique for medulloblastoma tumors in dosimetric comparison
Source: Radiat Oncol. 2020 Nov 5;15:256. doi: 10.1186/s13014-020-01690-5 (PMC7643335; doi:10.1186/s13014-020-01690-5)
Supplement: Supplementary file 4 — Additional file 4: Table S4. Dose statistics shown for the lungs for three VMAT methods. [file 13014_2020_1690_MOESM4_ESM.docx]

**Additional file 4:**

**Supplementary Table S4:** Dose statistics shown for the lungs for three VMAT methods.

| Technique | VMAT_AVD | VMAT_noAVD | VMAT_FullArc” |
| --- | --- | --- | --- |
| Lungs | Vol% | Vol% | Vol% |
| V5Gy | 65.2 [61.0-70.8] | 69.7 [64.6-74.0] | 92.1 [85.7-98.2] |
| V10Gy | 24.8 [20.9-28.9] | 19.9 [19.1-21.0] | 24.5 [20.9-27.8] |
| V20Gy | 2.0 [1.7-2.5] | 1.9 [1.6-2.1] | 1.9 [1.2-2.3] |

**Abbreviations:** V5Gy = Lungs volume receiving doses more than 5Gy; VMAT_AVD = Volumetric modulated arc therapy with avoidance sectors; VMAT_noAVD = VMAT without avoidance sectors; VMAT_FullArc = VMAT without 360 degrees rotation.
