# Supplementary material for: Pediatric craniospinal irradiation with a short partial-arc VMAT technique for medulloblastoma tumors in dosimetric comparison
Source: Radiat Oncol. 2020 Nov 5;15:256. doi: 10.1186/s13014-020-01690-5 (PMC7643335; doi:10.1186/s13014-020-01690-5)
Supplement: Supplementary file 5 — Additional file 5: Table S5. DVH comparison for three different VMAT setups. [file 13014_2020_1690_MOESM5_ESM.docx]

**Additional file 5:**

**Supplementary Figure S5:** DVH comparison for three different VMAT setups.

**Supplementary Figure S3**. Dose Volume Histogram comparison for three different VMAT techniques; “VMAT_AVD” trial (solid lines), the “noAVD” trial (dotted lines) and the “FullArc” trial (dashed lines).
